# Nutritional, environmental and economic impacts of ultra-processed food consumption in Australia

**DOI:** 10.1017/S136898002300232X

**Published:** 2023-12

**Authors:** Navoda Nirmani Liyanapathirana, Amanda Grech, Mengyu Li, Arunima Malik, Rosilene Ribeiro, Timur Burykin, Manfred Lenzen, David Raubenheimer

**Affiliations:** 1 ISA, School of Physics A28, The University of Sydney, Sydney, NSW 2006, Australia; 2 The School of Life and Environmental Sciences, Charles Perkins Centre, The University of Sydney, Sydney, NSW, Australia; 3 Discipline of Accounting, Business School, The University of Sydney, Sydney, NSW, Australia

**Keywords:** Input–output analysis, Nutritional geometry, Sustainability indicators, Ultra-processed foods

## Abstract

**Objective:**

To quantify the full life cycle impacts of ultra-processed foods (UPF) for key environmental, economic and nutritional indicators to identify trade-offs between UPF contribution to broad-scope sustainability.

**Design:**

Using 24-h dietary recalls along with an input–output database for the Australian economy, dietary environmental and economic impacts were quantified in this national representative cross-sectional analysis. Food items were classified into non-UPF and UPF using the NOVA system, and dietary energy contribution from non-UPF and UPF fractions in diets was estimated. Thereafter, associations between nutritional, environmental and economic impacts of non-UPF and UPF fractions of diets were examined using a multi-dimensional nutritional geometry representation.

**Setting:**

National Nutrition and Physical Activity Survey 2011–2012 of Australia.

**Participants:**

Respondents (n 5344) aged > 18 years with 1 d of 24-h dietary recall data excluding respondents with missing values and outlier data points and under reporters.

**Results:**

Australian diets rich in UPF were associated with reduced nutritional quality, high greenhouse gas emissions, energy use, and increased employment and income associated with the food supply chains. The environmental and economic impacts associated with the UPF portion of diets become more distinct when the diets are standardised to average protein recommendation.

**Conclusion:**

Increased consumption of UPF has socio-economic benefits, but this comes with adverse effects on the environment and public health. Consideration of such trade-offs is important in identifying policy and other mechanisms regarding UPF for establishing healthy and sustainable food systems.

Food consumption and dietary choices are key determinants of health and sustainability. A good deal of attention has been given to the effects of animal-based foods on health and sustainability^(1)^, but with the rapid global rise in the consumption of ultra-processed foods (UPF)^(2)^, there is an urgent need to understand their consequences for healthy and sustainable food systems.

UPF are industrial formulations of food substances with fats, sugar and salt added excessively during processing^(3)^. UPF also contain other additives such as flavours, flavour enhancers, colours and sweeteners that are not normally used in culinary preparations^(4)^. UPF are formulated to be competitively priced, highly palatable, and require minimum preparation compared with minimally processed and whole food alternatives^(5)^. UPF are often overconsumed because of their hyper-palatability, low satiety resulting from low levels of protein and fibre^(6)^ and aggressive marketing^(7)^.

The nutritional impacts of the increased consumption of UPF are relatively well studied^(8)^. There have, however, been very few quantitative analyses of the UPF contribution to environmental and economic sustainability^(9)^. Reviews on UPF and food systems sustainability have stated that UPF are associated with high greenhouse gas (GHG) emissions and water use and intensive agriculture/livestock practices resulting in deforestation, loss of biodiversity and high agrochemical use^(10,11)^. Efforts have been made to quantify the environmental impacts of excessively processed foods in Australia based on a nationally relevant food classification, which distinguishes between discretionary and core foods. These studies have shown that discretionary foods contribute 30–40 % of diet-related environmental impacts^(12,13)^. While most discretionary foods are also classified as UPF, foods such as commercial bread, flavoured yogurt and processed cheese are classified in the Australian dietary guidelines as core foods, and as UPF under NOVA, an international classification based on the degree of food processing^(14)^.

Such approaches, however, have not integrated nutritional, environmental and economic impacts of UPF to account for possible trade-offs between different dimensions of sustainability. An obvious win-win solution in terms of health and environment – discouraging the consumption of UPF – could have repercussions on other dimensions of sustainability. Furthermore, UPF utilise many recombinant ingredients and their full impact might not be captured within the system boundary of life cycle assessments (LCA)^(15)^. As such, the full life cycle impacts of UPF for multiple indicators across different domains of sustainability need to be quantified and their underlying relations need to be studied, understood and managed.

Our study aims firstly to quantify the full life cycle impacts of UPF and non-UPF fractions of diets for key environmental and economic indicators. Secondly, we aim to examine and demonstrate the potential of integrating input–output (IO) analysis with nutritional geometry to investigate the associations of nutritional, environmental, and economic indicators, and the relative contributions of UPF to diets in the Australian context. To the best of our knowledge, this is the first study to investigate the full environmental impacts of UPF and non-UPF fractions of diets and the relationships between nutritional, environmental, and economic sustainability of these foods to reveal possible trade-offs between indicators.

Our methodology is applicable to any country for which IO databases are available. However, the Australian food system is associated with the highest obesity rates among developed countries^(16)^ and significantly contributes to Australia being among the highest per capita carbon emitters in the world^(17)^. Australia is thus well suited for examining the utility of our approach and producing findings that might be equally applicable to other developed countries with similar consumption of UPF. To place our study in the international context, we used the NOVA food classification system, the most applied classification system in the scientific literature to classify foods based on the level of processing^(14)^. Our ultimate goal is to contribute towards objective approaches for quantifying the full contribution of UPF to food systems sustainability and identifying relations and trade-offs between UPF contribution to sustainability. Such information is important in formulating holistic and systematic solutions for healthy and sustainable concerns associated with UPF.

## Methodology

Recently, our team introduced a technique of nutrient-sensitive sustainability assessment method by integrating the macroeconomic concept of environmentally extended IO (EEIO) technique and the nutritional ecology concept of nutritional geometry^(18)^. In prior studies, this technique was used to assess the sustainability of current dietary patterns^(18)^ and macronutrient dietary recommendations of Australia^(18)^ in relation to macronutrient composition. In this paper, the nutrient-sensitive sustainability assessment method was modified to assess the associations between nutritional, environmental, and economic indicators, and the relative contributions of UPF to diets.

For that, the full life cycle impacts of UPF for environmental and economic indicators were first calculated using the EEIO technique, which is an extension of IO analysis. IO analysis is a macroeconomic concept introduced by Wassily Leontief for which he was awarded the Nobel Prize for Economic Science in 1973. IO analysis is a top-down economic technique that is based on interdependencies between various industries, and households in an economy in the form of supply and consumption of goods and services, formation of capital, and exchange of income and labour^(19)^. IO tables are the foundation for IO analysis representing the monetary flow of goods and services between industries and between industries, products, and consumers. The IO tables are an important element of national accounts data, and they can be constructed for a single region or a country, for example, an IO table of Australia, or multiple regions or countries, for example, an IO table for six states of Australia or an IO table for all countries in the world^(20)^. IO tables comprise an *N* × *N* intermediate transaction matrix **T** mapping the inter-sector transactions, an *N* × *M* final demand matrix **y** mapping supplies from sectors to final consumers and *K* × *M* value-added matrix **v** mapping primary inputs to sectors expressed in monetary units. In the application of EEIO to quantify the environmental and social impacts of monetary flows represented in IO tables, an *L* × *N* matrix **Q** representing the environmental and social impacts associated with sectors in physical units is used in addition to T, y and v tables.

Then, calculated environmental and economic indicators were integrated with a form of nutritional geometry called right-angled mixture triangles^(21)^ to assess the association between environmental and economic indicators and the relative contributions of UPF in diets. Nutritional geometry is a modelling technique that represents how the mixtures of nutrients (in this study, relative contribution from UPF and non-UPF to dietary energy) combine and affect the responses of interest (nutritional, environmental and economic indicators)^(6,21)^. The detailed methodology is described in the subsequent sections.

### Estimation of dietary intake data and calculation of the percentage of dietary energy from ultra-processed foods

We used the dietary intake data from the first day of two 24-h dietary recalls for 9341 Australian adults (*n* 9341) obtained from the most recent National Nutrition and Physical Activity Survey (NNPAS) – 2011–2013 conducted by the Australian Bureau of Statistics (ABS) in this study. Information on the sampling, demography and data collection procedure of the NNPAS is published^(18,22–24)^.

Then, to calculate the relative contribution of UPF and non-UPF in the diets of each respondent to dietary energy, the foods reported in the survey were classified into NOVA groups according to the prior study^(25)^. Then, to calculate the relative contribution of UPF and non-UPF in the diets of each respondent to dietary energy, the foods reported in the survey were classified into NOVA groups according to the prior study^(25)^. In the prior study, a group of registered nutritionists and dietitians used the Australian Food, Supplement and Nutrient Database (AUSNUT) 2011–2013 and ‘food details file’ that provides additional non-nutrition data such as origin (e.g. homemade or supermarket) of the food item and labelling data to classify the foods into NOVA categories. For example, Artisan and homemade bread were classified as processed, while mass-produced packaged bread were classified UPF based on the data in the ‘food details file’. The steps for classifying foods are elaborated^(25)^.

The NOVA food classification system classifies foods into four groups based on the degree of processing they had undergone^(4)^: group 1 includes unprocessed, fresh and foods that have undergone minimal processing such as freezing or drying, for example, vegetables, fruits, grains, meat, eggs and seafood. Group 2 includes culinary ingredients such as sugar, butter, yeast, salt and plant oils that are used for the preparation of group 1 foods. Group 3 includes foods that have undergone some processing or include ingredients from both group 1 and group 2, for example, canned or bottled fruits and vegetables, cured, smoked, salted, or sugared nuts and seeds, meats, cheese, and fresh bread. Group 4 includes foods that have been ultra-processed and include additives and preservatives that are not normally used in culinary formulations such as sweeteners, thickeners, hydrolysed proteins and maltodextrin. Examples of group 4 foods include soft drinks, packaged snack foods such as crisps, chocolates, margarine, reconstituted meat such as hot dogs, packaged instant noodles, breakfast cereals and alcoholic distilled drinks. Further details on the NOVA food classification system are published elsewhere^(4)^. After categorising the foods into four groups, the percentage of dietary energy from each food group was calculated for each respondent.

### Estimation of nutritional indicators

In our study, we used energy density and nutrient density as nutritional indicators. The energy density (kJ/100 g/d) is defined as the amount of dietary energy provided by 100 g of foods and beverages consumed per d and nutrient density (NRF 9.3/capita/d) is calculated using Nutrient Rich Food Index (NRF) 9.3^(26)^. Necessary nutritional information for the calculation was obtained from the AUSNUT 2011–2013 nutrient composition database^(27)^. Detailed calculations of energy density and nutrient density are published^(18)^.

### Estimation of environmental indicators

Environmental impacts associated with dietary intake were quantified using the EEIO technique. Four environmental indicators, namely GHG emissions (kg-CO2/capita/d), material flow (kg/capita/d), water use (kl/capita/d) and energy use (MJ/capita/d), were considered in our study. The indicators represent the full supply chain environmental impacts of dietary intake from all the upstream processes from the point of production to the point of purchase.

In this study, for the EEIO calculations, we derived a custom-built single-region supply-use table with forty-six economic sectors (see online Supplemental Table 1), including extensions for environmental indicators for the Australian economy for the year 2012 using the Australian Industrial Ecology Virtual Laboratory (IELab)^(28)^. Then, environmental intensities **q** and environmental multipliers **m** were calculated for each economic sector according to Leontief’s basic IO calculus. Further details on the calculation are published^(18)^. Then, the dietary intake of each respondent of NNPAS was categorised into forty-six sectors to match the economic sectors in the supply use table using the unique eight-digit ID assigned by ABS to group similar foods^(22)^. Further details on the categorisation of foods to forty-six economic sectors are published^(18)^.

Then, to integrate the dietary intake data in mass terms **y**_**m**_ with environmental multipliers **m** in dollar terms, **y**_**m**_ was converted into monetary units using basic (farm and factory gate) prices. Multiple linear regression was used to create a linear relationship between the dependent variable, basic prices, and independent variables, energy obtained from macronutrients, and NOVA categories (section 2 supplemental material). Then, the regression model was used to calculate the price of diet **π**. Dietary data in mass terms **y**_**m**_ were then converted into monetary terms as **y**_**$**_ = **y**_**m**_ **π**, and dietary environmental impacts **f** were calculated as **f** = **y**_**$**_ **m**.

Homogeneity is an important assumption of the EEIO technique where it is assumed that each sector in the economy produces a single homogenous good or service. This assumption becomes more accurate as the number of sectors increases, and ideally, there should be one sector associated with each unique product in the economy. However, brand- or individual-level sector resolution is impossible to realise by national statistical agencies in constructing IO tables, because the monetary transaction data available to them is restricted by the total input and sales bills of producers, and transaction origins and destinations are determined by data reconciliation. For the purposes of this study, we categorised non-processed or minimally processed foods and processed foods into separate sectors to differentiate food items based on the degree of processing. For example, highly processed vegetable products like vegetable chips and processed vegetable products such as canned vegetables were categorised under the vegetable product sector, while fresh and minimally processed vegetables were categorised under the vegetable sector.

The imports to and exports from Australia are represented with a ‘rest-of-world’ region in the IO model. All imports and exports are represented as a one-row/column item. Therefore, impacts associated with the goods or services imported to Australia are accounted for in the IO calculation. However, there is no country- or region-wise differentiation based on the origin of the good or service. Since the objective of the study is to evaluate the UPF contribution to environmental indicators not to evaluate regional differences of environmental impacts, we did not use a regional classification.

IO tables are for raw products, and the dietary data are for cooked food. Thus, in the calculation, it was assumed that there is no mass loss in the conversion of raw products for cooked foods. Additionally, estimating environmental impacts using the IO technique is different from using the process LCA technique. Process LCA only assesses selected processes in the supply chain, and its estimates tend to be lower than estimates from the IO approach^(29)^. Thus, the estimates from our study cannot be directly compared with the estimates from process LCA studies.

### Estimation of economic indicators

Income (AUD/capita/d), expenditure on food (AUD/capita/d), cost of dietary energy (AUD/100 kJ/d) and employment (full-time equivalent minutes/capita/d) were considered as economic indicators. The employment indicator represents the employment in all upstream processes from the point of production to the point of purchase in the supply chain of providing the dietary intake of an individual. The income indicator represents the wages and salaries received by households for their employment. The income and employment were calculated using the IO technique following the same procedure as described in section 2·3 and detailed calculations of economic indicators are published^(18)^.

### Construction of mixture triangles

The association between the indicators and relative contributions of UPF to diets was analysed using right-angled mixture triangles^(21)^. In the mixture triangles, axes represent the percentage of dietary energy from NOVA food categories (NOVA 1, the sum of NOVA 2 and 3 and NOVA 4) in diets and the response surfaces represent the variation of indicators against the relative contributions from NOVA food groups by way of a non-parametric thin-plate splines surface map. We allocated two separate axes for foods at two extreme ends in the classification of foods based on the degree of processing – X-axis representing the percentage of energy from NOVA 4 (UPF) foods, the Y-axis representing the percentage of energy from NOVA 1 food (fresh and minimally processed foods) and the rest of NOVA food groups were represented on one axis (hypotenuse). This highlights the variations of indicators based on the degree of processing from minimal processing to ultra-processing of foods.

**Fig. 1:**
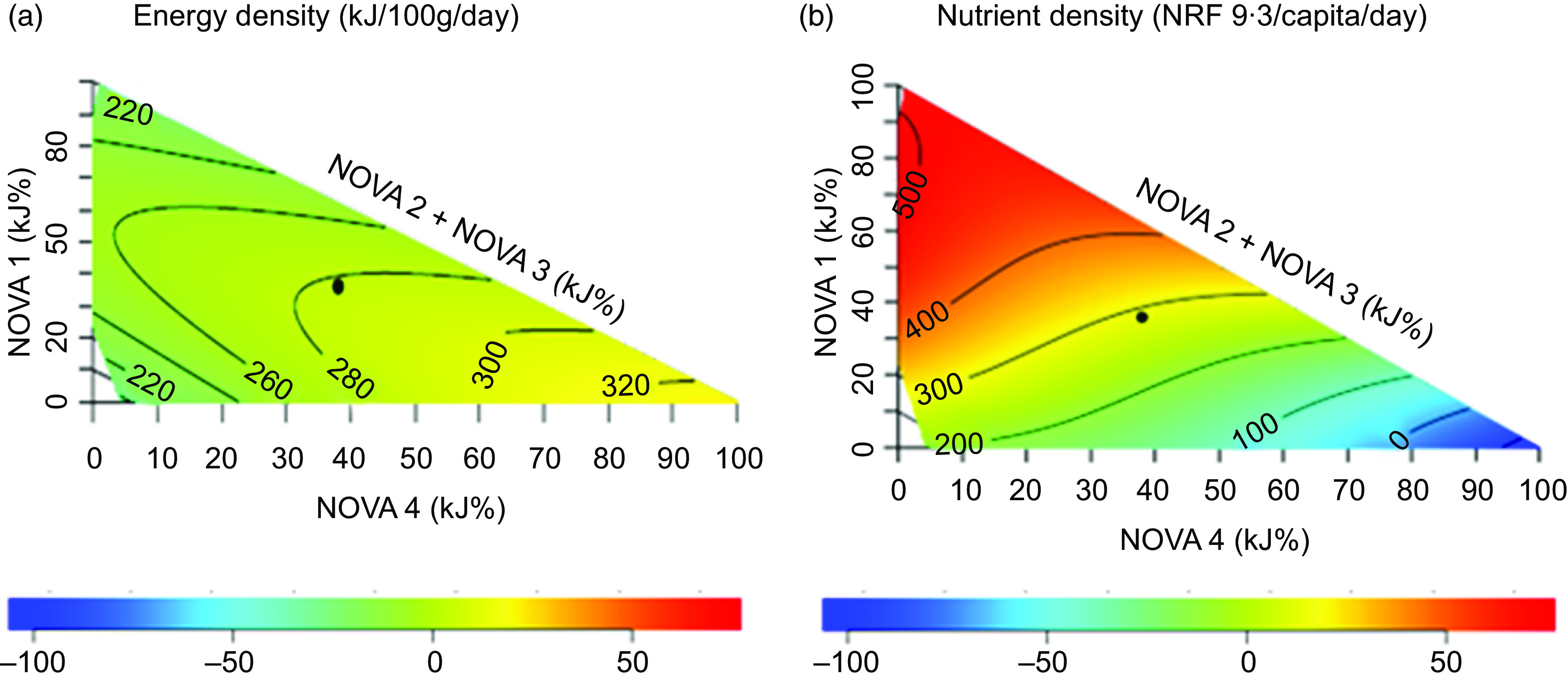
Variation of nutrient indicators against the percentage of dietary energy from NOVA food categories in diets of adult respondents in Australian National Nutrition and Physical Activity Survey 2011–2012 excluding respondents with missing values and outlier data points and under-reporters (*n* 5344). (a) Energy density; (b) nutrient density. The black dot in the mixture triangles indicates the average percentage of dietary energy from NOVA food categories in diets. The colours in the response surface indicate the departure (-100 % to +50 %; see the colour bar at the bottom of the mixture triangle) of observations from the mean of the plotted sample. NRF, nutrient-rich food index

**Fig. 2:**
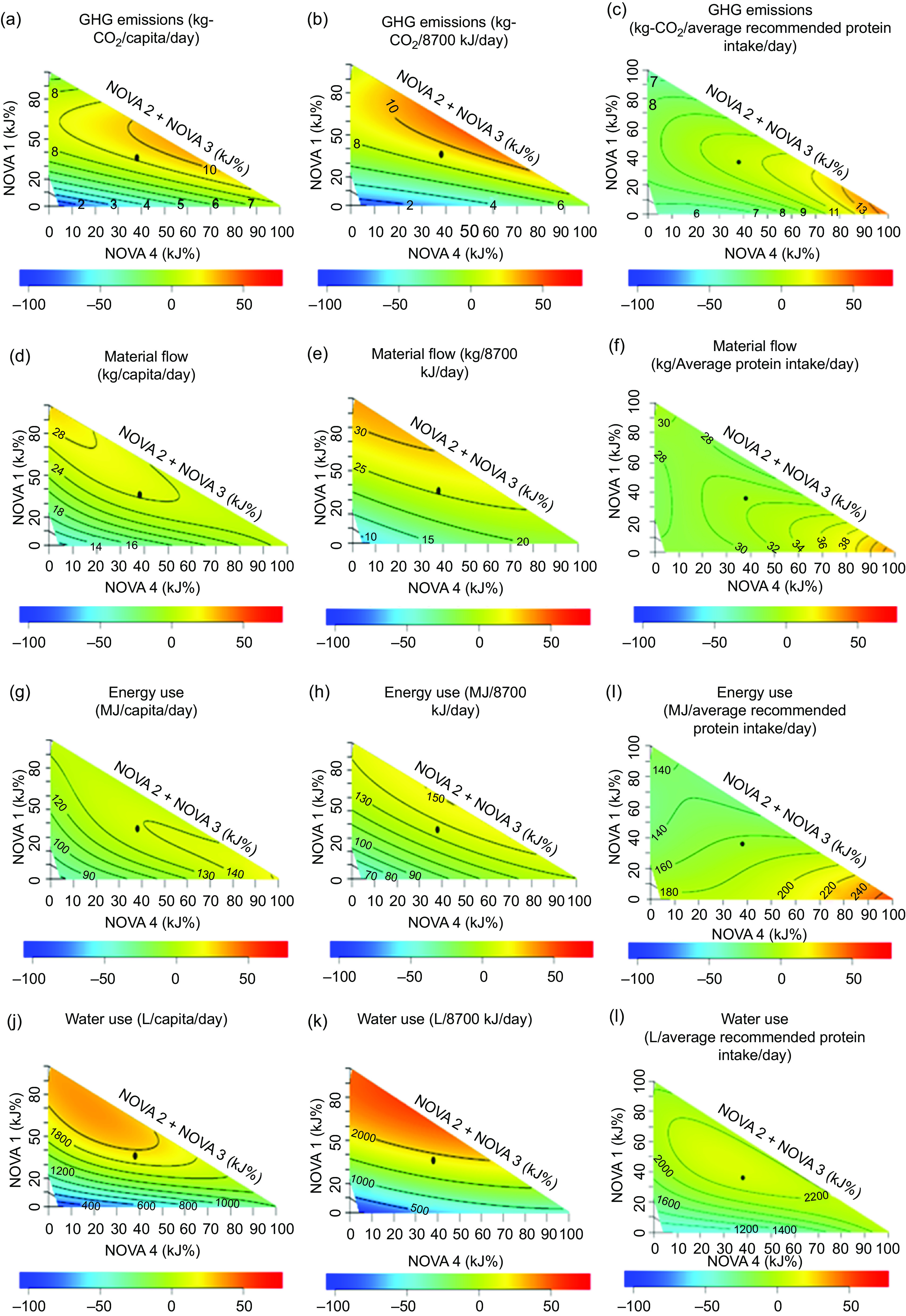
Variation of environmental indicators against the percentage of dietary energy from NOVA food categories in diets of adult respondents in Australian National Nutrition and Physical Activity Survey 2011–2012 excluding respondents with missing values and outlier data points and under-reporters (*n* 5344). (a) GHG emissions per capita; (b) GHG emission per 8700 kJ; (c) GHG emissions per average recommended protein intake; (d) material flow per capita; (e) material flow per 8700 kJ; (f) material flow per average recommended protein intake; (g) energy use per capita; (h) energy use per 8700 KJ; (i) energy use per average recommended protein intake; (j) water use per capita; (k) water use per 8700 kJ and (l) water use per average recommended protein intake. The black dot in the mixture triangles indicates the average percentage of dietary energy from NOVA food categories in diets. The colours in the response surface indicate the departure (-100 % to +50 %; see the colour bar at the bottom of the mixture triangle) of observations from the mean of the plotted sample. GHG, greenhouse gas.

**Fig. 3:**
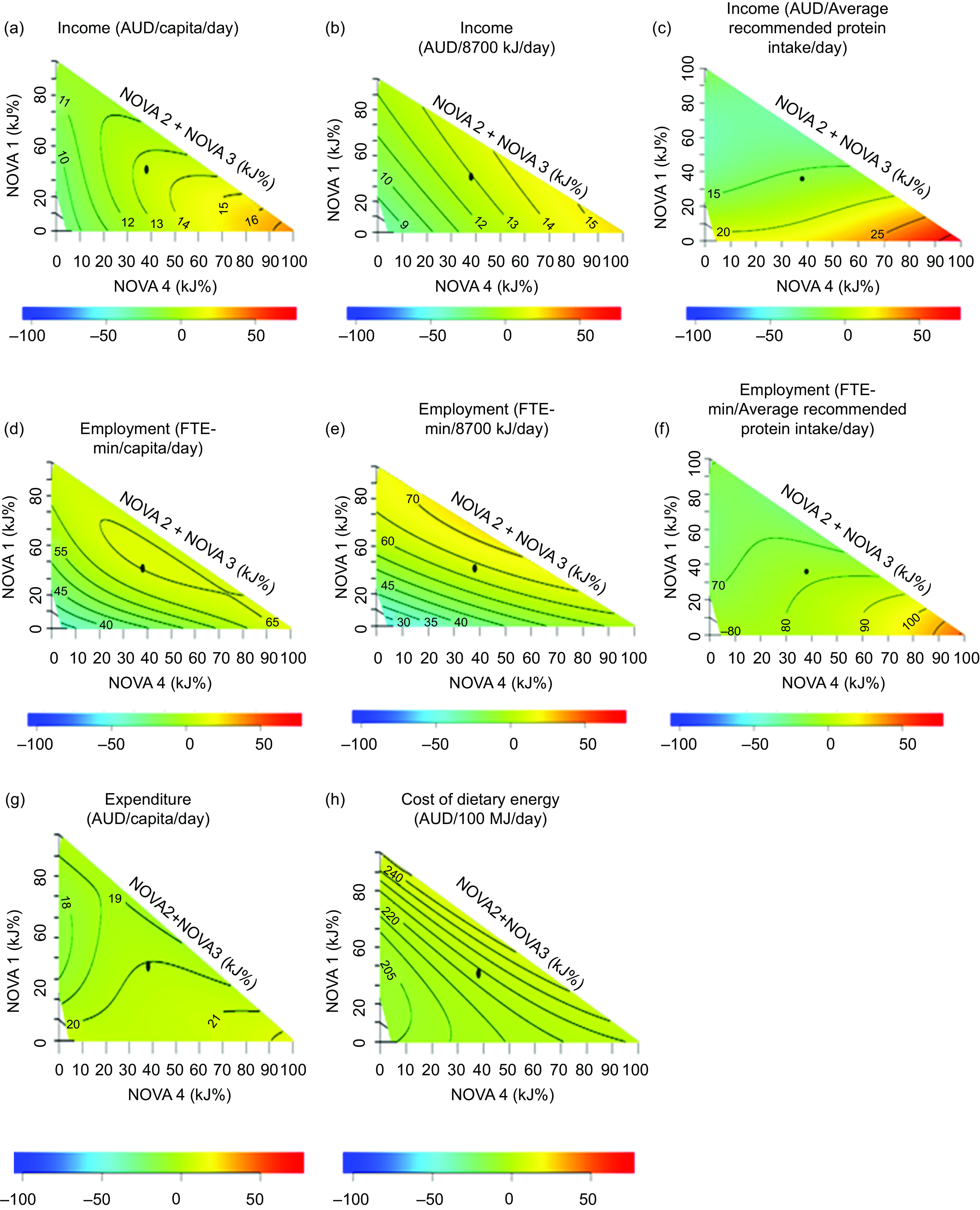
Variation of economic indicators against the percentage of dietary energy from NOVA food categories in diets adult respondents in Australian National Nutrition and Physical Activity Survey 2011–2012 excluding respondents with missing values and outlier data points and under-reporters (*n* 5344). (a) Income per capita; (b) income per 8700 kJ; (c) income per average recommended protein intake; (d) employment per capita; (e) employment per 8700 kJ; (f) employment per average recommended protein intake; (g) expenditure per capita and (h) cost of dietary energy. The black dot in the mixture triangles indicates the average percentage of dietary energy from NOVA food categories in diets. The colours in the response surface indicate the departure (-100 % to +50 %; see the colour bar at the bottom of the mixture triangle) of observations from the mean of the plotted sample. AUD, Australian dollars; FTE-min, full-time equivalent minutes

Scheffe’s mixture models were used to estimate response surfaces against the percentage of energy from NOVA food categories. Scheffe’s model with the lowest Akaike information criterion was used to estimate the response surface. The colours in the response surface indicate the departure (-100 % to +50 %; see the colour bar in Figs. 1 to 3) of observations from the mean of the plotted sample. The detailed explanation on the construction of mixture triangles is described elsewhere^(6,21)^. The mixture triangles were plotted using R studio version 1.2.5033.

For one set of mixture triangles, response surfaces represented per capita economic and environmental indicators without any standardisations to characterise the variations of impacts of dietary intakes as reported by the respondents in the survey against non-UPF and UPF fractions in diets. Then, we plotted two additional series of mixture triangles, in which response surfaces represented indicators standardised to average recommended energy intake (8700 kJ) and average recommended protein intake (20 % of dietary energy from proteins, being midway within the recommended 15–25 % range^(30)^), respectively, relative to energy intake reported by the respondents. These analyses enable us to examine what the impacts would be if Australians ate to recommended energy and protein intakes and compare those with the impacts of dietary intakes as reported by the respondents. Through our analyses, we focused on the broader implications of the relationship between impacts associated with UPF and non-UPF fractions and macronutrient recommendations.

Outliers and under-reporters were removed before plotting the mixture triangles and performing associated statistical analysis to eliminate the effect of outliers and under-reporters. Prior publications^(18)^ provide further details on criteria used to exclude outliers and under-reporters in this study. The ABS provided weightage for each respondent to estimate the population level variable using the responses for individuals in the survey. However, the association of indicators with relative proportions of UPF in diets was analysed at the individual diet level. As such, no weightage was applied in the calculation of indicators. The averages of the indicators were expressed for the all the respondents. Hence, the weighted average and standard deviation of indicators (Table 1) were calculated using the product-sum method using the weightage provided for each respondent. All data manipulations and statistical analyses were performed using MATLAB R2018b.

**Table 1:** Weighted average and sd of intakes, percentage of dietary energy from NOVA food categories in the diet and nutritional, environmental, and economic indicators for the adult respondents in Australian National Nutrition and Physical Activity Survey 2011–2012 excluding respondents with missing values and outlier data points and under-reporters (*n* 5344)

	Indicators	Weighted* average	sd
Intakes	Daily dietary energy intake per person (kJ)	9131	2618
	Daily dietary intake per person (g)	3473	1255
	Daily protein intake per person (g)	93·9	35·3
Percentage of dietary energy from NOVA food categories in diet	Percentage of dietary energy from NOVA 1 foods in the diet (%)	35·8	18·0
	Percentage of dietary energy from NOVA 2 and 3 foods in the diet (%)	25·5	15·3
	Percentage of dietary energy from NOVA 4 foods in the diet (%)	38·8	20·7
Nutritional indicators	Energy density (kJ/100 g)	281·9	86·2
	Nutrient density (NRF 9·3 %)	283·7	259·1
Environmental indicators	GHG emission (kg-CO_2_/capita/d)	8·8	5·8
	GHG emission (kg-CO_2_/102 g proteins/d)	9·9	5·8
	Material flow (kg/capita/d)	25·2	8·2
	Material flow (kg/102 g proteins/d)	30·3	13·0
	Energy use (MJ/capita/d)	134·3	38·1
	Energy use (MJ/102 g proteins/d)	167·1	83·9
	Water use (l/capita/d)	1753	884·4
	Water use (l/102 g proteins/d)	2089	1195
Economic indicators	Expenditure on food (AUD/capita/d)	19·9	5·6
	Cost of dietary energy (AUD/100 MJ)	221·9	37·89
	Income (AUD/capita/d)	13·2	4·0
	Income (AUD/102 g proteins/d)	15·9	6·9
	Employment (FTE-min/capita/d)	62·6	18·1
	Employment (FTE-min/102 g proteins/d)	74·9	28·8

NRF, nutrient-rich food index; GHG, greenhouse gas; AUD, Australian dollars; FTE-min, full-time equivalent minutes.

* Weight assigned to each respondent of the National Nutrition and Physical Activity Survey by the Australian Bureau of Statistics at the person level to estimate that the total population was used as the weighting variable.

## Results

The variations of the nutritional, environmental (per capita, per average recommended energy intake and average recommended protein intake) and economic (per capita, per average recommended energy intake and per average recommended protein intake) indicators against the percentage of dietary energy from NOVA food categories in diets as visualised in mixture triangles are shown in Figs. 1 to 3, respectively.

### Nutritional indicators

We observed that whilst the diets rich in UPF (NOVA4) are energy-dense (Fig. 1(a)), diets rich in fresh and unprocessed foods (NOVA 1) are most nutrient-dense (Fig. 1(b)). Furthermore, we found that on average Australian adults obtained more of dietary energy from UPF than any other food category – about 39 % (Table 1).

### Environmental indicators

The GHG emissions per capita (Fig. 2(a)) and per 8700 kJ (Fig. 2(b)) surface plots showed a maximum at about 50 % NOVA 4, about 50 % NOVA 1, and about 0 % NOVA 2 and 3, indicating that UPF (NOVA 4) and fresh and minimally processed foods (NOVA 1) contribute significantly and equally to GHG emissions. The energy use per capita (Fig. 2(g)) and per 8700 kJ (Fig. 2(h)) surface plots showed a maximum for diets rich in UPF. Material flow per capita (Fig. 2(d)) and water use per capita (Fig. 2(g)) surface plots showed a maximum for diets rich in fresh and minimally processed foods (NOVA 1). Material flow (Fig. 2(e)) and water use (Fig. 2(h)) per 8700 surface plots showed similar variations to that of their per capita plots. All the environmental indicators per average recommended protein intake (Fig. 2(c), (f), (i) and (l)) increased with the increase of energy from NOVA 4 foods (UPF).

### Economic indicators

The per capita (Fig. 3(d)) and per 8700 kJ (Fig. 3(e)) employment surface plots showed that high employment is associated with the supply chain of diets rich in both NOVA 4 and NOVA 1 foods with low NOVA 2 and 3 foods. As for income, high per capita (Fig. 3(a)) and per 8700 kJ (Fig. 3(b)) incomes were associated with the supply chain of diets rich in UPF. The expenditure (Fig. 3(g)) and cost of dietary energy (Fig. 3(h)) surface plots showed no apparent change in proportional contributions to energy from each NOVA food category. Once the diets were standardised to the average protein requirement, both income (Fig. 3(c)) and employment (Fig. 3(f)) showed a more prominent increase with the increase in the percentage of energy from UPF.

## Discussion

### Nutritional indicators

The removal of fibre and the addition of fats and sugar during the processing of UPF increases its energy density and dilutes nutrient density^(3)^, contributing to the observed variation of nutritional indicators. Additionally, the use of refined ingredients in their manufacture reduces their vitamin and mineral content compared to fresh and minimally processed foods^(31)^. Our findings indicate that on average Australian adults obtained about 40 % of dietary energy from UPF. Increased consumption of UPF is associated with many health problems such as obesity, type 2 diabetes, hypertension, CVD, cancers and gastrointestinal diseases such as irritable bowel syndrome^(32)^. Our findings reiterated the relatively well-established fact that UPF have adverse consequences for health and nutrition.

### Environmental indicators

According to our results, diets that contain equal proportions of UPF and fresh and minimally processed foods showed high per capita GHG emissions. It is well established that animal-based foods are associated with high GHG emissions^(33)^. The fresh and minimally processed foods portion of diets includes animal-based foods that likely contribute to high GHG emissions associated with the fresh and minimally processed food portion in diets. As for high GHG emissions associated with the UPF portion of diets, prior studies have reported that high GHG emissions of diets rich in UPF are mostly related to the emissions associated with additional processing and packaging rather than its raw materials^(34,35)^. Refining a product to the ready-to-eat stage at the point of purchase requires additional energy, and UPF such as potato chips, instant coffee, crisps, chocolate and commercial bread are among the most energy-intensive food products^(34,36)^. High energy use also contributes to the increase in GHG emissions, and moreover, additional energy required for additional processing and packaging is the most likely explanation for high energy use associated with diets rich in UPF.

Diets rich in fresh and minimally processed foods are associated with high per capita material flow and water use most likely because fresh and minimally processed foods utilise more raw materials and water in all upstream processes from point of production to point of purchase in their supply chain. Consistent with this is the observation that fruit and vegetable sectors that contain fresh and minimally processed foods showed a high contribution to water use and material flow compared to other sectors (see online Supplemental Table 2).

Our findings indicate that variation of environmental impacts against UPF and non-UPF does not change significantly if Australians consume to average recommended energy intake in comparison with variations of per capita environmental indicators. More significantly, environmental impacts associated with diets rich in UPF become more prominent once standardised to average protein recommendation. A likely explanation for this is that humans regulate protein intake more tightly than energy intake, leading to passive variation in energy based on protein percentage (‘protein leverage’)^(37)^. Two implications arise from this: (i) protein recommendations are adhered to more strictly than energy recommendations, making a protein-focused correction more realistic; and (ii) UPF are typically low in protein, impacting environmental indicators due to impacts associated with the production and processing of surplus energy eaten on these foods^(38,39)^.

### Economic indicators

High per capita employment associated with the supply chain of diets rich in both NOVA 4 and NOVA 1 foods with less NOVA 2 and 3 foods indicates that the same level of per capita employment is associated with the production and purchase of UPF and fresh foods. But, the sectors contributing significantly to employment in diets rich in UPF and diets rich in fresh and minimally processed foods were not the same. ‘Vegetables’, ‘fruits and nuts’, ‘dairy’ and ‘beverage’ sectors showed high contributions to employment in diets rich in fresh and minimally processed food, whilst ‘beverages (soft drinks)’ and ‘cereal and cereal-based products’ sectors showed high contributions to employment in diets rich in UPF (see online Supplemental Fig. 10). However, high per capita incomes were associated with the supply chain of diets rich in UPF. A possible explanation for this observation is that wages in the manufacturing sector are higher than wages in the agriculture sector^(40)^. This explanation is further supported by the fact that ‘beverages’, ‘dairy’, and ‘cereal and cereal-based products’ showed high contributions to income compared to other sectors (see online Supplemental Table 2).

The expenditure and cost of dietary energy did not show substantial variations with the percentages of energy from NOVA categories. However, the cost of dietary energy and expenditure is estimated using a regression model based on export prices (section 2 supplemental material) not market prices. Export prices are farm gate/basic prices (excluding taxes and government subsidies). It is possible that if these indicators were based on market prices, the inferences could be different from ours.

Similar to environmental indicators, both income and employment per average recommended energy intake showed a similar variation to per capita indicators and a more prominent increase with the increase of the percentage of energy from UPF when standardised to average protein recommendation. As discussed in section 4·2, for diets rich in UPF higher intakes are required to meet consumers’ need for protein, and increased consumption of UPF to meet the protein requirement resulted in diets rich in UPF being associated with even higher income and employment.

Previous studies have associated poor nutrient-quality diets with high environmental impacts (GHG emissions, water use, cropland scarcity use, etc.) through analysis done using either LCA or IO technique^(12,41,42)^. Our findings showing that diets rich in UPF, which have poor nutrient quality, are associated with high GHG emissions and energy use are consistent with previously reported findings. Our findings also showed that it is not environmentally sustainable if Australians meet their protein recommendation through current dietary patterns as the adverse environmental impacts associated with UPF-rich diets increased further when standardised to average protein recommendation. As such, the dietary recommendations need to advise the consumption of protein-dense, plant-based fresh foods to meet the protein recommendations to minimise adverse dietary environmental impacts. Additionally, our study focused on multiple environmental, economic and nutrition indicators enabling a more comprehensive assessment of impact from UPF on the environment and food systems and made it possible to evaluate trade-offs between indicators addressing one of the key limitations mentioned in previous studies^(12,41)^. However, we emphasise that this is not a complete measure of the environmental and economic impacts of UPF. Deforestation, loss of biodiversity, land use, food affordability using market prices and a range of other impacts must also be considered to fully understand the environmental and economic implications of UPF.

The use of dietary intake data as reported in NNPAS in the analysis provides the actual representation of the current consumption patterns in Australia as opposed to a mathematically modelled scenario. However, Australia is the third fastest-growing vegan market worldwide^(43)^, and the dietary transitions to protein-dense yet ultra-processed plant-based meat substitutes over time could potentially alter the environmental impacts associated with UPF and variation of environmental indicators standardised to daily protein requirements^(44)^. Modelling the impacts of such dietary transitions is outside the scope of our analysis. Given the growing popularity of plant-based meat substitutes, however, future studies investigating the environmental impacts of ultra-processed plant-based meat substitutes and their relation to protein satiety are needed to understand the implications of such dietary transitions. We only used the dietary recalls from the first day of interviews in this study. Even though 1-d recall does not capture the usual intake of an individual as well as would the average of two 1-d recalls, they do capture the food items consumed by respondents and accurately reflect environmental and economic impacts for all combinations of foods reported on a single day including extreme values which together comprise the population’s mean intake^(45)^.

Furthermore, under-reporting is an inherent limitation in self-reported dietary data, which was addressed in our analysis by excluding under-reporters. The NRF model used for the calculation of nutrient density is based mostly on the nutrients to limit: fats/saturated fats, Na and added sugar; thus, low nutrient densities are highly correlated with energy density^(46)^. Thus, this model has limitations for ranking the diets based on quality. The use of a diet-based nutrient indicator such as the hybrid NRF model^(46)^ or the Australian Healthy Eating Index^(47)^ could equally be used in future studies.

## Conclusions

We showed that consumption of UPF for meeting nutritional requirements, particularly protein requirements, leads to adverse environmental impacts. This is mainly because UPF contain low levels of protein; hence, they need to be consumed in higher amounts for reaching recommended protein intake. We further showed that when quantified using per capita dietary intakes, the environmental impacts of UPF are less evident. This study further highlights the trade-offs between UPF environmental, social and economic impacts. For example, promoting the sale of UPF may increase national economic benefits, as measured in per capita employment and per capita income terms, but this comes with adverse effects on the environment and public health. Using our findings, policymakers can examine all positive and negative impacts of UPF to gain a holistic view of what policy approach is best suited to addressing the negative impacts of UPF.

An important consideration when examining policy responses is the costs associated with nutrition-related health diseases such as poor diets and obesity. Poor dietary choices and the health costs of obesity in Australia have caused annual production losses (based on the human capital approach) of $591 million and between $840 million and $14·9 billion, respectively^(48)^. It is predicted, based on the trajectory of consumption of UPF, that these healthcare costs will continue to increase. This reveals that short-term national economic benefits such as higher per capita incomes resulting from increased consumption of UPF may be reduced by the public expenditure costs on health problems relating to poor nutrition. Additionally, with studies now indicating the environmental impacts of healthcare systems^(49)^, it is paramount that the environmental impacts of UPF do not create cascading environmental impacts elsewhere. Further studies on health-related environmental costs of UPF will strengthen the evidence base for policy formulation regarding UPF.

## Supporting information

Liyanapathirana et al. supplementary material Liyanapathirana et al. supplementary material

## Supplementary material

For supplementary material accompanying this paper visit https://doi.org/10.1017/S136898002300232X
