# Supplementary material for: Nutritional, environmental and economic impacts of ultra-processed food consumption in Australia
Source: Public Health Nutr. 2023 Oct 26;26(12):3359–69. doi: 10.1017/S136898002300232X (PMC10755453; doi:10.1017/S136898002300232X)
Supplement: Liyanapathirana et al. supplementary material [file S136898002300232Xsup001.docx]

**Supplementary Material**

Table of Contents

[1. Economic sectors 2](#_Toc134478276)

[2. Price regression model construction 3](#_Toc134478277)

[2.1. Multiple linear regression for data variables including the outliers 4](#_Toc134478278)

[2.1.1. Without the intercept 4](#_Toc134478279)

[2.1.2. With the intercept 5](#_Toc134478280)

[2.2. Multiple linear regression for data variables excluding the outliers 5](#_Toc134478281)

[2.2.1. Without the intercept 5](#_Toc134478282)

[2.2.2. With the intercept 6](#_Toc134478283)

[2.3. Weighted multiple linear regression for data variables including the outliers 6](#_Toc134478284)

[2.3.1. Daily dietary intake as the weighting variable 6](#_Toc134478285)

[2.3.2. Square root of daily dietary intake as the weighting variable 7](#_Toc134478286)

[2.3.3. Energy intake as the weighting variable 8](#_Toc134478287)

[2.3.4. Square root of energy intake as the weighting variables 8](#_Toc134478288)

[2.4. Weighted multiple linear regression for data variables excluding the outliers 9](#_Toc134478289)

[2.4.1. Daily dietary intake as the weighting variable 9](#_Toc134478290)

[2.4.2. Square root of daily dietary intake as the weighting variable 10](#_Toc134478291)

[2.4.3. Energy intake as the weighting variable 11](#_Toc134478292)

[2.4.4. Square root of energy intake as the weighting variables 12](#_Toc134478293)

[3. Sector-wise analysis 14](#_Toc134478298)

[3.1. Contribution to environmental and economic indicators calculated using the input-output technique 14](#_Toc134478299)

[3.2. Sector-wise contribution to dietary intake for diets rich in UPFs (>95% of energy from NOVA 4), NOVA 2 and 3 (>95% of energy from NOVA 2 and 3), and NOVA 1 (>95% of energy from NOVA 1) 15](#_Toc134478308)

[3.3. Sector-wise contribution to diets rich in UPFs (>95% of energy from NOVA 4), NOVA 2 and 3 (>95% of energy from NOVA 2 and 3), and NOVA 1 (>95% of 17](#_Toc134478309)

# Economic sectors

Supplemental Table 1- Economic sectors in the supply-use table of Australia used in the calculation of dietary environmental and economic indicators using environmentally extended input-output analysis

| No | Sector |
| --- | --- |
| 1 | Sheep and goats |
| 2 | Beef |
| 3 | Poultry |
| 4 | Pigs |
| 5 | Other livestock and meat |
| 6 | Sheep meat |
| 7 | Beef meat |
| 8 | Poultry meat |
| 9 | Pork |
| 10 | Fish |
| 11 | Crustacean and molluscs |
| 12 | Dairy products |
| 13 | Eggs |
| 14 | Jams, marmalade and honey |
| 15 | Other animal products |
| 16 | Plant nurseries |
| 17 | Vegetables |
| 18 | Nuts |
| 19 | Fruit |
| 20 | Coffee, tea and spices |
| 21 | Wheat |
| 22 | Cereal grains |
| 23 | Wheat flour |
| 24 | Grain flours and products |
| 25 | Soybeans, ground nuts, copra, seeds |
| 26 | Sugar |
| 27 | Other roots and fibres and extracts |
| 28 | Animal-based oils and fats |
| 29 | Plant-based oils and fats |
| 30 | Meat products |
| 31 | Fish products |
| 32 | Crustacean and mollusc products |
| 33 | Sugar products |
| 34 | Cocoa and chocolate |
| 35 | Cereal products |
| 36 | Vegetable products |
| 37 | Fruit products |
| 38 | Extracts and ice cream |
| 39 | Waters and soft drinks |
| 40 | Alcoholic beverages |
| 41 | Animal food |
| 42 | Tobacco |
| 43 | Other manufacturing |
| 44 | Restaurants |
| 45 | Catering |
| 46 | Other services |
| 47 | Exports* |

*All imports and exports are represented as a one-row item. Therefore, impacts associated with the goods or services imported to Australia are accounted for in the input-output calculation. However, there is no country-wise differentiation based on the origin of the good or service.

# Price regression model construction

Export data for 6357 economic sectors of Harmonized commodity description and coding system (HSCPC) for the year 2015 was obtained from the United Nations International Trade Statistics Database (UN COMTRADE). The export price (USD per kg) for 6357 economic sectors was calculated for 221 partnering countries for the reporting country, Australia. Row median for a HSCPC sector indicates the price paid by the consumer in Australia, for a 1kg of product in the HSCPC sector produced in Australia. For example, the row median for the beef sector gives the price per 1kg of beef produced in Australia. Thus, it was assumed that all the food items consumed in Australia are produced within Australia itself. The HSCPC sectors were linked with the nutrients and NOVA categories of foods in the AUSNUT database by way of a concordance matrix between HSCPC sectors and AUSNUT foods.

At first, the linearity between the median export price and NOVA categories, the percentage of energy obtained from macronutrients (protein, carbohydrate, and fat) were tested using scatter plots (Supplementary Figure 1).


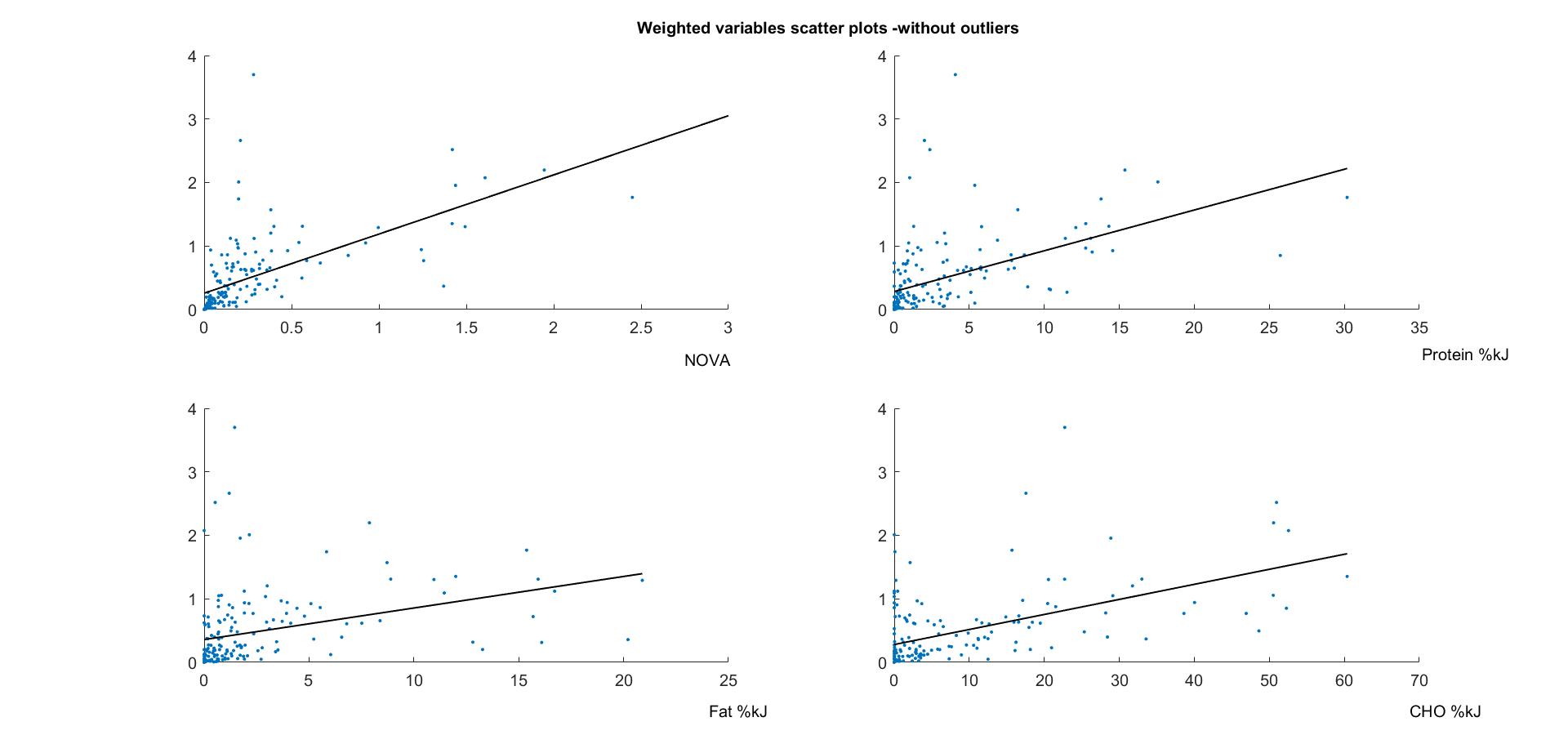


Price

Price

Price

Price

Supplementary Figure 1 – Scatter plots between median export price and NOVA categories, percentage of energy obtained from macronutrients (protein, carbohydrate, and fat)

Then, multiple linear regression analysis was performed to model the relationship between the NOVA categories, the percentage of energy obtained from macronutrients (protein, carbohydrate, and fat), and the median export price, where the median export price was considered as the response variable and NOVA categories and percentage of energy from macronutrients were considered as explanatory variables at different scenarios as follows:

1. Including outliers (The data points situated more than 1.5 interquartile ranges above the upper quartile (75%) or below the lower quartile (25%) were considered as outliers)
   1. No weighing variable – with and without intercept
   2. Daily dietary intake as the weighting variable – with and without intercept
   3. Square root of daily dietary intake as the weighting variable – with and without intercept
   4. Energy intake as the weighting variable – with and without intercept
   5. Square root of energy intake as the weighting variables – with and without intercept
2. Excluding outliers
   1. No weighing variable – with and without intercept
   2. Daily dietary intake as the weighting variable – with and without intercept
   3. Square root of daily dietary intake as the weighting variable – with and without intercept
   4. Energy intake as the weighting variable – with and without intercept
   5. Square root of energy intake as the weighting variables – with and without intercept

After that, the statistical significance of the model was tested using t-tests. If the t-statistical value is greater than the t-critical value (table S1), it was concluded that there is statistical evidence of a linear relationship between the response variable and explanatory variable at that level of a confidence interval.

| Confidence interval | t-critical values | |  |
| --- | --- | --- | --- |
|  | Degrees of freedom = 162 | Degrees of freedom = 266 |  |
| 90% | 1.654314 | 1.650559 | * |
| 95% | 1.974716 | 1.968855 | ** |
| 99% | 2.606518 | 2.594298 | *** |
| 99.90% | 3.351595 | 3.327198 | **** |

Supplemental Table 2 - t-critical values at different confidence intervals

All the regression analysis and associated statistical analysis were performed using MATLAB R2018b software. The resultant models and associated statistical values are as follows:

## Multiple linear **regression for data variables** including the outliers

### Without the intercept

*Linear regression model:*

*y = x1 + x2 + x3 + x4*

*y= Median export price (AUD/kg)*

*x1= NOVA*

*x2 = Protein (%kJ)*

*x3 = Fat (%kJ)*

*x4 = CHO (%kJ)*

*Estimated Coefficients:*

|  | *Estimate* | *Standard deviation* | *t-statistical value* | *p value* |
| --- | --- | --- | --- | --- |
| *x1* | *10.838* | *13.974* | *0.77556* | *0.4387* |
| *x2* | *0.99273** | *0.56574* | *1.7547* | *0.080455* |
| *x3* | *-0.18602* | *0.53057* | *-0.35061* | *0.72616* |
| *x4* | *-0.33365* | *0.41917* | *-0.79597* | *0.42676* |

*Number of observations: 270, Error degrees of freedom: 266*

*Root Mean Squared Error: 257*

*R-squared: 0*.*0140*

*R-squared adjusted: 0.0029*

### With the intercept

*Linear regression model:*

*y = 1 + x1 + x2 + x3 + x4*

*y= Median export price (AUD/kg)*

*x1= NOVA*

*x2 = Protein (%kJ)*

*x3 = Fat (%kJ)*

*x4 = CHO (%kJ)*

*Estimated Coefficients:*

|  | *Estimate* | *Standard deviation* | *t-statistical value* | *p value* |
| --- | --- | --- | --- | --- |
| *Intercept* | *0* | *0* | *NaN* | *NaN* |
| *x1* | *10.838* | *13.974* | *0.77556* | *0.4387* |
| *x2* | *0.99273** | *0.56574* | *1.7547* | *0.080455* |
| *x3* | *-0.18602* | *0.53057* | *-0.35061* | *0.72616* |
| *x4* | *-0.33365* | *0.41917* | *-0.79597* | *0.42676* |

*Number of observations: 270, Error degrees of freedom: 266*

*Root Mean Squared Error: 257*

*R-squared: 0.014*

*Adjusted R-Squared: 0.0029*

*F-statistic vs. constant model: 1.26, p-value = 0.288*

## Multiple linear regression for data variables excluding the outliers

### Without the intercept

*Linear regression model:*

*y = x1 + x2 + x3 + x4*

*y= Median export price (AUD/kg)*

*x1= NOVA*

*x2 = Protein (%kJ)*

*x3 = Fat (%kJ)*

*x4 = CHO (%kJ)*

*Estimated Coefficients:*

|  | *Estimate* | *Standard deviation* | *t-statistical value* | *p value* |
| --- | --- | --- | --- | --- |
| *x1* | *0.013553* | *0.23032* | *0.058844* | *0.95315* |
| *x2* | *0.043882***** | *0.010738* | *4.0864* | *6.8766e-05* |
| *x3* | *0.059296***** | *0.008387* | *7.0699* | *4.3876e-11* |
| *x4* | *0.032947***** | *0.0058982* | *5.586* | *9.6205e-08* |

*Number of observations: 166, Error degrees of freedom: 162*

*Root Mean Squared Error: 3.06*

*R-squared: 0.0685*

*R-squared adjusted: 0.0513*

### With the intercept

*Linear regression model:*

*y = 1 + x1 + x2 + x3 + x4*

*y= Median export price (AUD/kg)*

*x1= NOVA*

*x2 = Protein (%kJ)*

*x3 = Fat (%kJ)*

*x4 = CHO (%kJ)*

*Estimated Coefficients:*

|  | *Estimate* | *Standard deviation* | *t-statistical value* | *p value* |
| --- | --- | --- | --- | --- |
| *Intercept* | *0* | *0* | *NaN* | *NaN* |
| *x1* | *0.013553* | *0.23032* | *0.058844* | *0.95315* |
| *x2* | *0.043882***** | *0.010738* | *4.0864* | *6.8766e-05* |
| *x3* | *0.059296***** | *0.008387* | *7.0699* | *4.3876e-11* |
| *x4* | *0.032947***** | *0.0058982* | *5.586* | *9.6205e-08* |

*Number of observations: 166, Error degrees of freedom: 162*

*Root Mean Squared Error: 3.06*

*R-squared: 0.0685*

*Adjusted R-Squared: 0.0513*

*F-statistic vs. constant model: 3.97, p-value = 0.00915*

## Weighted multiple linear regression for data variables including the outliers

### Daily dietary intake as the weighting variable

#### With the intercept

*Linear regression model:*

*y = 1 + x1 + x2 + x3 + x4*

*y= Median export price (AUD/kg)*

*x1= NOVA*

*x2 = Protein (%kJ)*

*x3 = Fat (%kJ)*

*x4 = CHO (%kJ)*

*Estimated Coefficients:*

|  | *Estimate* | *Standard deviation* | *t-statistical value* | *p value* |
| --- | --- | --- | --- | --- |
| *Intercept* | *4473.3* | *3903.3* | *1.146* | *0.25282* |
| *x1* | *0.71725* | *1.9013* | *0.37724* | *0.7063* |
| *x2* | *0.028811* | *0.17984* | *0.1602* | *0.87284* |
| *x3* | *0.044856* | *0.14296* | *0.31376* | *0.75395* |
| *x4* | *-0.010193* | *0.079548* | *-0.12813* | *0.89814* |

*Number of observations: 270, Error degrees of freedom: 265*

*Root Mean Squared Error: 5.76e+04*

*R-squared: 0.0175,*

*Adjusted R-Squared: 0.00268*

*F-statistic vs. constant model: 1.18, p-value = 0.32*

#### Without the intercept

*Linear regression model:*

*y = x1 + x2 + x3 + x4*

*y= Median export price (AUD/kg)*

*x1= NOVA*

*x2 = Protein (%kJ)*

*x3 = Fat (%kJ)*

*x4 = CHO (%kJ)*

*Estimated Coefficients:*

|  | *Estimate* | *Standard deviation* | *t-statistical value* | *p value* |
| --- | --- | --- | --- | --- |
| *x1* | 0.26302 | 1.8606 | 0.14136 | 0.88769 |
| *x2* | 0.053577 | 0.17864 | 0.29991 | 0.76448 |
| *x3* | 0.072632 | 0.14098 | 0.51521 | 0.60683 |
| *x4* | 0.0099572 | 0.077626 | 0.12827 | 0.89803 |

*Number of observations: 270, Error degrees of freedom: 266*

*Root Mean Squared Error: 5.77e+04*

### Square root of daily dietary intake as the weighting variable

#### With the intercept

*Linear regression model:*

*y = 1 + x1 + x2 + x3 + x4*

*y= Median export price (AUD/kg)*

*x1= NOVA*

*x2 = Protein (%kJ)*

*x3 = Fat (%kJ)*

*x4 = CHO (%kJ)*

*Estimated Coefficients:*

|  | *Estimate* | *Standard deviation* | *t-statistical value* | *p value* |
| --- | --- | --- | --- | --- |
| *Intercept* | *282.48* | *335.1* | *0.84299* | *0.4* |
| *x1* | *5.3946* | *7.9236* | *0.68083* | *0.49657* |
| *x2* | *0.56843* | *0.53437* | *1.0637* | *0.28841* |
| *x3* | *-0.34183* | *0.47245* | *-0.72353* | *0.46999* |
| *x4* | *-0.25262* | *0.31511* | *-0.80168* | *0.42345* |

*Number of observations: 270, Error degrees of freedom: 265*

*Root Mean Squared Error: 3.83e+03*

*R-squared: 0.00759,*

*Adjusted R-Squared: -0.00739*

*F-statistic vs. constant model: 0.507, p-value = 0.731*

#### Without the intercept

*Linear regression model:*

*y = x1 + x2 + x3 + x4*

*y= Median export price (AUD/kg)*

*x1= NOVA*

*x2 = Protein (%kJ)*

*x3 = Fat (%kJ)*

*x4 = CHO (%kJ)*

*Estimated Coefficients:*

|  | *Estimate* | *Standard deviation* | *t-statistical value* | *p value* |
| --- | --- | --- | --- | --- |
| *x1* | *3.3682* | *7.5461* | *0.44635* | *0.65571* |
| *x2* | *0.72334* | *0.50151* | *1.4423* | *0.15039* |
| *x3* | *-0.21864* | *0.44903* | *-0.48691* | *0.62672* |
| *x4* | *-0.14233* | *0.28651* | *-0.49678* | *0.61975* |

*Number of observations: 270, Error degrees of freedom: 266*

*Root Mean Squared Error: 3.83e+03*

### Energy intake as the weighting variable

#### With the intercept

*Linear regression model:*

*y = 1 + x1 + x2 + x3 + x4*

*y= Median export price (AUD/kg)*

*x1= NOVA*

*x2 = Protein (%kJ)*

*x3 = Fat (%kJ)*

*x4 = CHO (%kJ)*

*Estimated Coefficients:*

|  | *Estimate* | *Standard deviation* | *t-statistical value* | *p value* |
| --- | --- | --- | --- | --- |
| *Intercept* | *13311* | *20694* | *0.64321* | *0.52064* |
| *x1* | *-2.0824* | *1.3806* | *-1.5083* | *0.13267* |
| *x2* | *0.086249* | *0.058897* | *1.4644* | *0.14427* |
| *x3* | *0.13228*** | *0.052705* | *2.5097* | *0.01268* |
| *x4* | *0.14447* | *0.065967* | *2.19* | *0.029398* |

*Number of observations: 270, Error degrees of freedom: 265*

*Root Mean Squared Error: 3.15e+05*

*R-squared: 0.266,*

*Adjusted R-Squared: 0.254*

*F-statistic vs. constant model: 24, p-value = 6.31e-17*

#### Without the intercept

*Linear regression model:*

*y = x1 + x2 + x3 + x4*

*y= Median export price (AUD/kg)*

*x1= NOVA*

*x2 = Protein (%kJ)*

*x3 = Fat (%kJ)*

*x4 = CHO (%kJ)*

*Estimated Coefficients:*

|  | *Estimate* | *Standard deviation* | *t-statistical value* | *p value* |
| --- | --- | --- | --- | --- |
| *x1* | *-2.2221* | *1.3619* | *-1.6316* | *0.10394* |
| *x2* | *0.090436* | *0.058471* | *1.5467* | *0.12313* |
| *x3* | *0.13901**** | *0.051599* | *2.6939* | *0.00075103* |
| *x4* | *0.15082**** | *0.065153* | *2.3148* | *0.021385* |

*Number of observations: 270, Error degrees of freedom: 266*

*Root Mean Squared Error: 3.15e+05*

### Square root of energy intake as the weighting variables

#### With the intercept

*Linear regression model:*

*y = 1 + x1 + x2 + x3 + x4*

*y= Median export price (AUD/kg)*

*x1= NOVA*

*x2 = Protein (%kJ)*

*x3 = Fat (%kJ)*

*x4 = CHO (%kJ)*

*Estimated Coefficients:*

|  | *Estimate* | *Standard deviation* | *t-statistical value* | *p value* |
| --- | --- | --- | --- | --- |
| *Intercept* | *977.72* | *826.58* | *1.1828* | *0.23793* |
| *x1* | *4.4788* | *6.5377* | *0.68507* | *0.4939* |
| *x2* | *0.24003* | *0.34611* | *0.69351* | *0.4886* |
| *x3* | *-0.1457* | *0.25561* | *-0.57* | *0.56916* |
| *x4* | *-0.23901* | *0.31684* | *-0.75436* | *0.4513* |

*Number of observations: 270, Error degrees of freedom: 265*

*Root Mean Squared Error: 8.87e+03*

*R-squared: 0.00556,*

*Adjusted R-Squared: -0.00945*

*F-statistic vs. constant model: 0.37, p-value = 0.83*

#### Without the intercept

*Linear regression model:*

*y = x1 + x2 + x3 + x4*

*y= Median export price (AUD/kg)*

*x1= NOVA*

*x2 = Protein (%kJ)*

*x3 = Fat (%kJ)*

*x4 = CHO (%kJ)*

*Estimated Coefficients:*

|  | *Estimate* | *Standard deviation* | *t-statistical value* | *p value* |
| --- | --- | --- | --- | --- |
| *x1* | *0.92676* | *5.8116* | *0.15947* | *0.87342* |
| *x2* | *0.40828* | *0.31577* | *1.293* | *0.19715* |
| *x3* | *-0.0003689* | *0.22431* | *-0.0016447* | *0.99869* |
| *x4* | *-0.034433* | *0.26567* | *-0.12961* | *0.89698* |

*Number of observations: 270, Error degrees of freedom: 266*

*Root Mean Squared Error: 8.88e+03*

## Weighted multiple linear regression for data variables excluding the outliers

### Daily dietary intake as the weighting variable

#### With the intercept

*Linear regression model:*

*y = 1 + x1 + x2 + x3 + x4*

*y= Median export price (AUD/kg)*

*x1= NOVA*

*x2 = Protein (%kJ)*

*x3 = Fat (%kJ)*

*x4 = CHO (%kJ)*

*Estimated Coefficients:*

|  | *Estimate* | *Standard deviation* | *t-statistical value* | *p value* |
| --- | --- | --- | --- | --- |
| *Intercept* | *154.28***** | *41.796* | *3.6912* | *0.00030508* |
| *x1* | *0.42271**** | *0.15025* | *2.8134* | *0.0055138* |
| *x2* | *0.033256***** | *0.0090281* | *3.6837* | *0.00031356* |
| *x3* | *0.0086235* | *0.010181* | *0.84702* | *0.39824* |
| *x4* | *0.010532**** | *0.0038973* | *2.7023* | *0.0076248* |

*Number of observations: 166, Error degrees of freedom: 161*

*Root Mean Squared Error: 404*

*R-squared: 0.461,*

*Adjusted R-Squared: 0.447*

*F-statistic vs. constant model: 34.4, p-value = 1.01e-20*

#### Without the intercept

*Linear regression model:*

*y = x1 + x2 + x3 + x4*

*y= Median export price (AUD/kg)*

*x1= NOVA*

*x2 = Protein (%kJ)*

*x3 = Fat (%kJ)*

*x4 = CHO (%kJ)*

*Estimated Coefficients:*

|  | *Estimate* | *Standard deviation* | *t-statistical value* | *p value* |
| --- | --- | --- | --- | --- |
| *x1* | *0.3813**** | *0.15556* | *2.4511* | *0.015303* |
| *x2* | *0.039866***** | *0.0091871* | *4.3393* | *2.5073e-0.5* |
| *x3* | *0.018482** | *0.0102* | *1.8119* | *0.071852* |
| *x4* | *0.01489***** | *0.0038562* | *3.8612* | *0.00016276* |

*Number of observations: 166, Error degrees of freedom: 162*

*Root Mean Squared Error: 420*

*R-squared: 0.5378*

*R-squared adjusted: 0.4790*

### Square root of daily dietary intake as the weighting variable

#### With the intercept

*Linear regression model:*

*y = 1+ x1 + x2 + x3 + x4*

*y= Median export price (AUD/kg)*

*x1= NOVA*

*x2 = Protein (%kJ)*

*x3 = Fat (%kJ)*

*x4 = CHO (%kJ)*

*Estimated Coefficients:*

|  | *Estimate* | *Standard deviation* | *t-statistical value* | *p value* |
| --- | --- | --- | --- | --- |
| *Intercept* | *14.555**** | *4.3366* | *3.3564* | *0.00098531* |
| *x1* | *0.37891** | *0.20661* | *1.8339* | *0.068508* |
| *x2* | *0.042774***** | *0.0096934* | *4.4128* | *1.862e-05* |
| *x3* | *0.016251* | *0.011484* | *1.4151* | *0.15898* |
| *x4* | *0.0081558* | *0.0057274* | *1.424* | *0.15639* |

*Number of observations: 166, Error degrees of freedom: 161*

*Root Mean Squared Error: 30.6*

*R-squared: 0.243,*

*Adjusted R-Squared: 0.224*

*F-statistic vs. constant model: 12.9, p-value = 3.88e-09*

#### Without the intercept

*Linear regression model:*

*y = x1 + x2 + x3 + x4*

*y= Median export price (AUD/kg)*

*x1= NOVA*

*x2 = Protein (%kJ)*

*x3 = Fat (%kJ)*

*x4 = CHO (%kJ)*

*Estimated Coefficients:*

|  | *Estimate* | *Standard deviation* | *t-statistical value* | *p value* |
| --- | --- | --- | --- | --- |
| *x1* | *0.35395** | *0.21292* | *1.6624* | *0.098366* |
| *x2* | *0.05388***** | *0.0093953* | *5.7348* | *4.6647e-0.8* |
| *x3* | *0.032691**** | *0.010712* | *3.0519* | *0.0026585* |
| *x4* | *0.017386**** | *0.0051807* | *3.356* | *0.0009852* |

*Number of observations: 166, Error degrees of freedom: 162*

*Root Mean Squared Error: 31.6*

*R-squared: 0.3639*

*R-squared adjusted: 0.3521*

### Energy intake as the weighting variable

#### With the intercept

*Linear regression model:*

*y = 1+ x1 + x2 + x3 + x4*

*y= Median export price (AUD/kg)*

*x1= NOVA*

*x2 = Protein (%kJ)*

*x3 = Fat (%kJ)*

*x4 = CHO (%kJ)*

*Estimated Coefficients:*

|  | *Estimate* | *Standard deviation* | *t-statistical value* | *p value* |
| --- | --- | --- | --- | --- |
| *Intercept* | *1153.2* | *811.88* | *1.4204* | *0.15744* |
| *x1* | *0.10924* | *0.23474* | *0.46537* | *0.6423* |
| *x2* | *0.026315* | *0.016621* | *1.5833* | *0.11532* |
| *x3* | *0.055338***** | *0.0079928* | *6.9235* | *9.9471e-11* |
| *x4* | *0.02741**** | *0.0090469* | *3.0298* | *0.0028521* |

*Number of observations: 166, Error degrees of freedom: 161*

*Root Mean Squared Error: 6.71e+03*

*R-squared: 0.452,*

*Adjusted R-Squared: 0.438*

*F-statistic vs. constant model: 33.2, p-value = 3.49e-20*

#### Without the intercept

*Linear regression model:*

*y = x1 + x2 + x3 + x4*

*y= Median export price (AUD/kg)*

*x1= NOVA*

*x2 = Protein (%kJ)*

*x3 = Fat (%kJ)*

*x4 = CHO (%kJ)*

*Estimated Coefficients:*

|  | *Estimate* | *Standard deviation* | *t-statistical value* | *p value* |
| --- | --- | --- | --- | --- |
| *x1* | *0.045638* | *0.23115* | *0.19744* | *0.84373* |
| *x2* | *0.034174*** | *0.015722* | *2.1737* | *0.031183* |
| *x3* | *0.059753***** | *0.0073866* | *8.0893* | *1.3358e-13* |
| *x4* | *0.033417***** | *0.0080225* | *4.1654* | *5.0404e-05* |

*Number of observations: 166, Error degrees of freedom: 162*

*Root Mean Squared Error: 6.74e+03*

*R-squared: 0.4885*

*R-squared adjusted: 0.4790*

### Square root of energy intake as the weighting variables

#### With the intercept

*Linear regression model:*

*y = 1+ x1 + x2 + x3 + x4*

*y= Median export price (AUD/kg)*

*x1= NOVA*

*x2 = Protein (%kJ)*

*x3 = Fat (%kJ)*

*x4 = CHO (%kJ)*

*Estimated Coefficients:*

|  | *Estimate* | *Standard deviation* | *t-statistical value* | *p value* |
| --- | --- | --- | --- | --- |
| *Intercept* | *36.337* | *27.401* | *1.3261* | *0.18668* |
| *x1* | *0.095181* | *0.26014* | *0.36588* | *0.71493* |
| *x2* | *0.033105** | *0.017709* | *1.8694* | *0.063384* |
| *x3* | *0.055124***** | *0.0097768* | *5.6382* | *7.5338e-08* |
| *x4* | *0.023827*** | *0.010875* | *2.1909* | *0.029894* |

*Number of observations: 166, Error degrees of freedom: 161*

*Root Mean Squared Error: 141*

*R-squared: 0.268,*

*Adjusted R-Squared: 0.25*

*F-statistic vs. constant model: 14.8, p-value = 2.72e-10*

#### Without the intercept

*Linear regression model:*

*y = x1 + x2 + x3 + x4*

*y= Median export price (AUD/kg)*

*x1= NOVA*

*x2 = Protein (%kJ)*

*x3 = Fat (%kJ)*

*x4 = CHO (%kJ)*

*Estimated Coefficients:*

|  | *Estimate* | *Standard deviation* | *t-statistical value* | *p value* |
| --- | --- | --- | --- | --- |
| *x1* | *0.027869* | *0.25574* | *0.10897* | *0.91336* |
| *x2* | *0.047419**** | *0.014073* | *3.3696* | *0.0009409* |
| *x3* | *0.062186***** | *0.008218* | *7.5671* | *2.7153e-12* |
| *x4* | *0.034001***** | *0.0077268* | *4.4004* | *1.9527e-05* |

*Number of observations: 166, Error degrees of freedom: 162*

*Root Mean Squared Error: 142*

*R-squared: 0.3111*

*R-squared adjusted: 0.2984*

Based on the statistical significance and the R-square adjusted values, the model derived under sub heading 2.4.1.2. was chosen among all the models to estimate the price of the AUSNUT foods.

The equation below shows the derived regression model:

$$\mathbf{Price}= \mathbf{0.3813*NOVA+}\boldsymbol{0.039866*Protein\%kJ+ 0.018482*Fat\%kJ+ 0.01489*CHO\%kJ}$$

The percentage of energy from macronutrients from each food item in AUSNUT food database and NOVA category of each food items weighted with the daily dietary intake of the food item were substituted to the above model one food at a time to calculate the price per kg of each food items. Then, the gram value of dietary intake each food items were converted to dollar values using the estimated price and the dietary intake per each respondent in the survey was calculated after the conversion.

# Sector-wise analysis

## Contribution to environmental and economic indicators calculated using the input-output technique

Supplemental Table 2 – Contribution to total GHG emissions, Material flow, Energy use, Water use, Employment, and Income by food-related sectors (The food-related sectors were separated from 46 economic sectors and similar sectors were aggregated into one sector. The original sector numbers (as numbered in Supplemental Table 2) are indicated in brackets)

| Food related sectors | GHG emissions (kg-CO2e) | Material flow (kg) | Energy use (MJ) | Water use (L) | Employment (FTE-min) | Income (AUD) |
| --- | --- | --- | --- | --- | --- | --- |
| Beverages (Non-alcoholic/alcoholic) (39,40) | 5138 | 43323 | 252947 | 2089 | 81715 | 30509 |
| Dairy (12) | 7895 | 5459 | 52003 | 3176 | 19464 | 4991 |
| Cereals and cereal-based products (21-24, 35) | 561 | 6051 | 43415 | 259 | 13199 | 4867 |
| Confectionary (26,33, 34, 38) | 275 | 3217 | 10045 | 202 | 2981 | 1673 |
| Other plant-based products (14, 20, 29, 36, 37) | 160 | 4028 | 10593 | 158 | 4283 | 1150 |
| Vegetables (17, 25, 27) | 1524 | 12254 | 41992 | 2761 | 19030 | 600 |
| Fruits and nuts (18, 19) | 774 | 9109 | 32299 | 3383 | 15662 | 462 |
| Meat (Poultry, Beef, Lamb, pork and other livestock meat) (1-9) | 12125 | 3542 | 16691 | 529 | 9510 | 353 |
| Other animal-based products (13, 15, 28, 30) | 462 | 426 | 1792 | 30 | 937 | 287 |
| Seafoods (10, 11, 31, 32) | 84 | 781 | 2343 | 215 | 667 | 71 |

## Sector-wise contribution to dietary intake for diets rich in UPFs (>95% of energy from NOVA 4), NOVA 2 and 3 (>95% of energy from NOVA 2 and 3), and NOVA 1 (>95% of energy from NOVA 1)

Supplemental Figure 3 - Sector-wise analysis of dietary intake of diets rich in NOVA 1 foods

Supplemental Figure 4 - Sector-wise analysis of dietary intake of diets rich in UPF (NOVA 4 foods)

Supplemental Figure 5 - Sector-wise analysis of dietary intake of diets rich in NOVA 2 and 3 foods

##
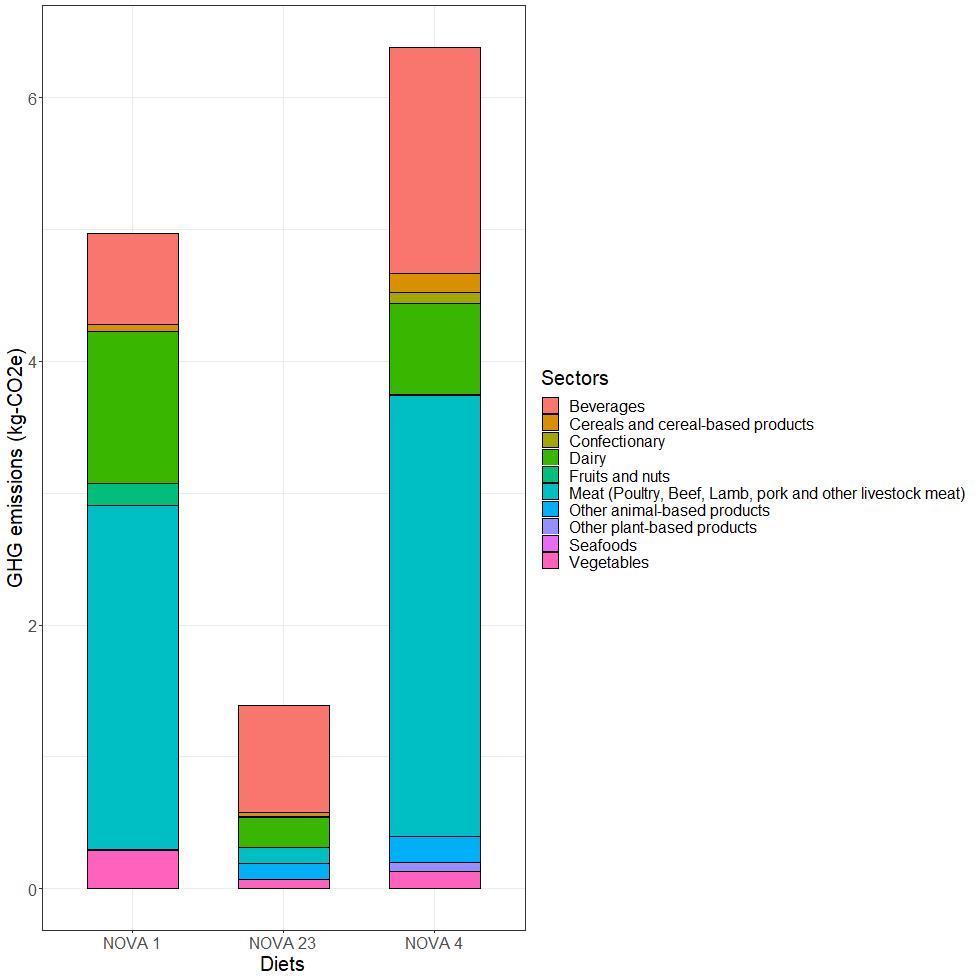
Sector-wise contribution to diets rich in UPFs (>95% of energy from NOVA 4), NOVA 2 and 3 (>95% of energy from NOVA 2 and 3), and NOVA 1 (>95% of

Supplemental Figure 6 - Sector-wise contribution to GHG emissions for diets rich in NOVA 1 (>95% energy from NOVA 1), NOVA 23 (>95% energy from NOVA 2 and 3), and NOVA 4 (>95% energy from NOVA 4)


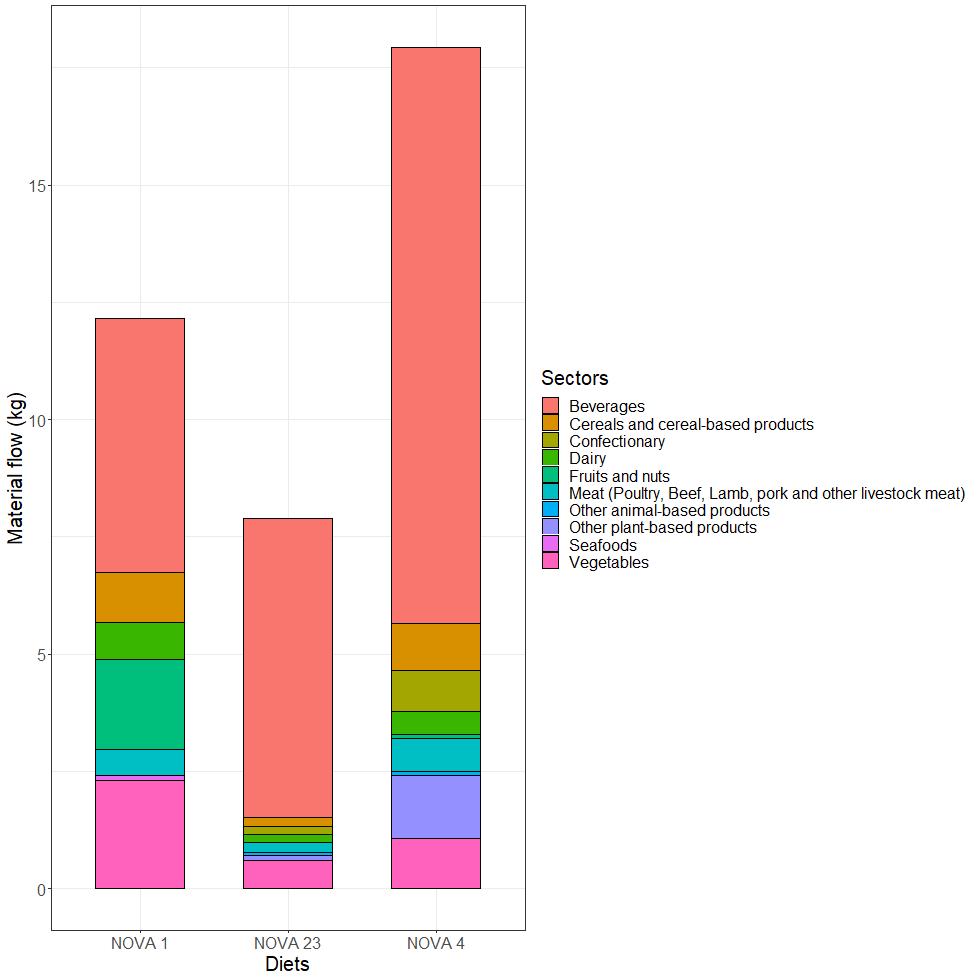


Supplemental Figure 7 - Sector-wise contribution to material flow for diets rich in NOVA 1 (>95% energy from NOVA 1), NOVA 23 (>95% energy from NOVA 2 and 3), and NOVA 4 (>95% energy from NOVA 4)


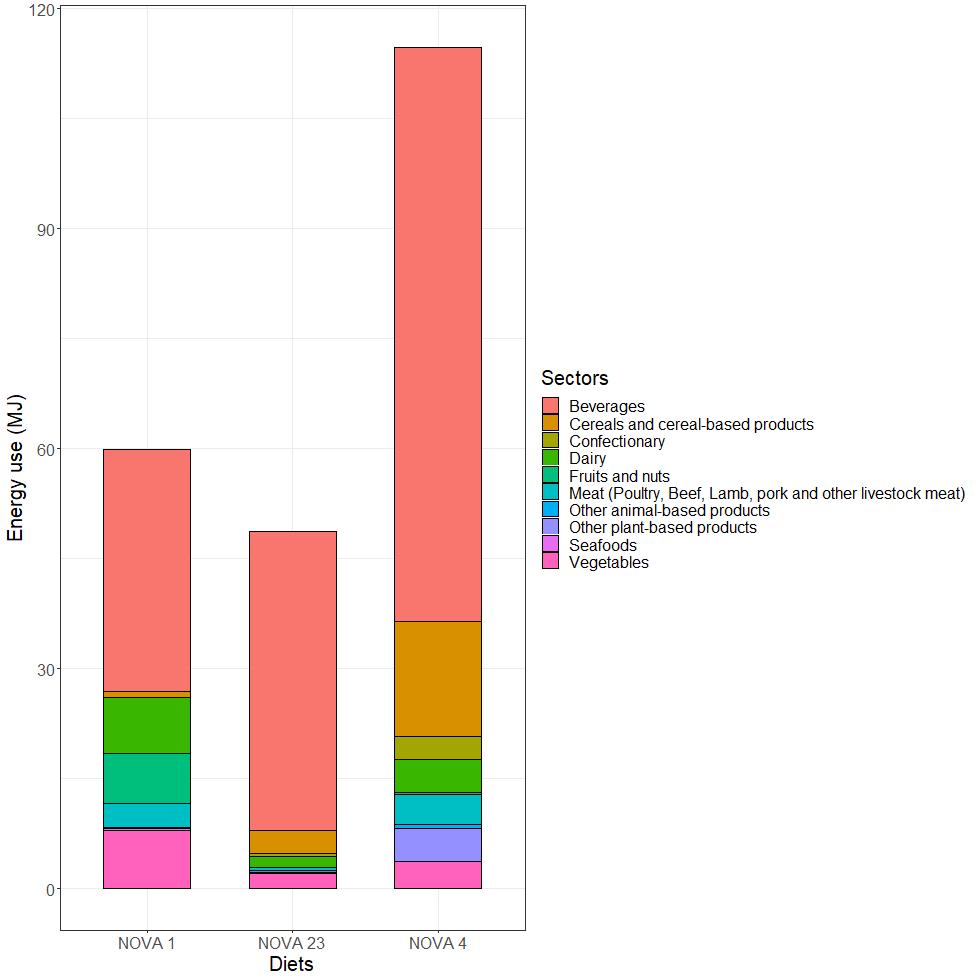


Supplemental Figure 8 - Sector-wise contribution to energy use for diets rich in NOVA 1 (>95% energy from NOVA 1), NOVA 23 (>95% energy from NOVA 2 and 3), and NOVA 4 (>95% energy from NOVA 4)


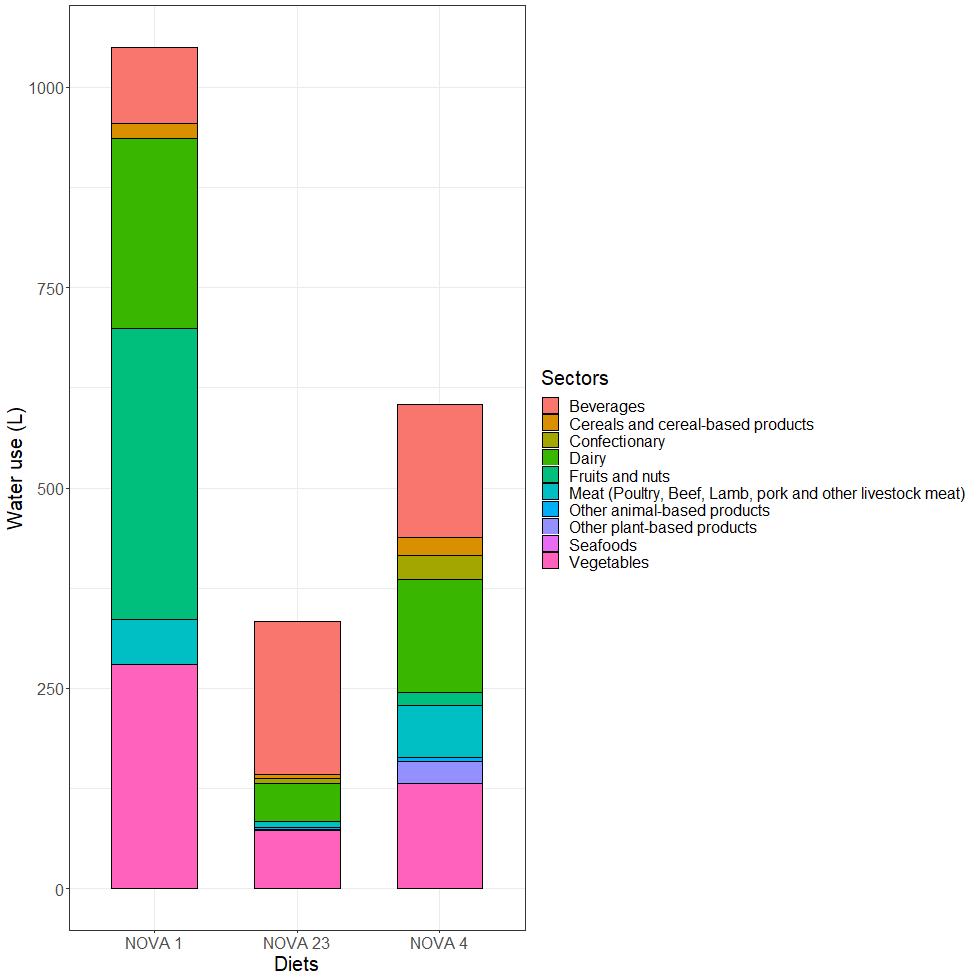


Supplemental Figure 9 - Sector-wise contribution to water use for diets rich in NOVA 1 (>95% energy from NOVA 1), NOVA 23 (>95% energy from NOVA 2 and 3), and NOVA 4 (>95% energy from NOVA 4)


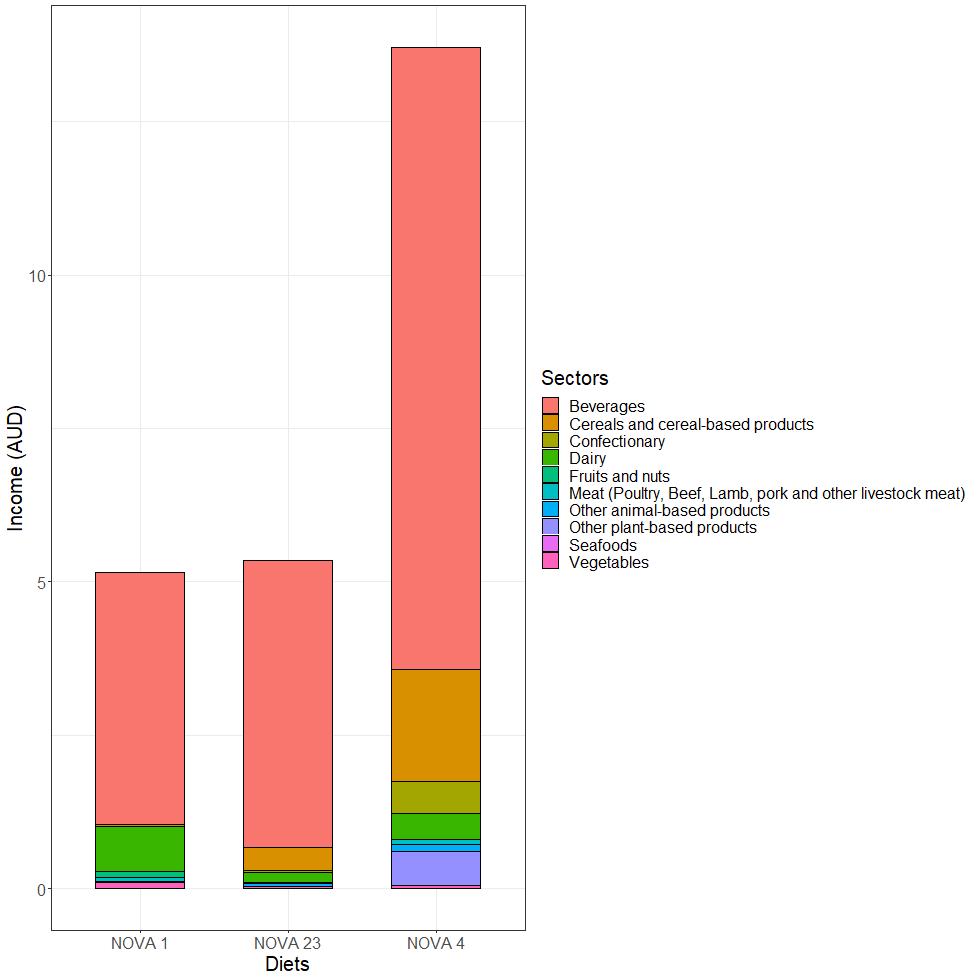


Supplemental Figure 10 - Sector-wise contribution to income for diets rich in NOVA 1 (>95% energy from NOVA 1), NOVA 23 (>95% energy from NOVA 2 and 3), and NOVA 4 (>95% energy from NOVA 4)


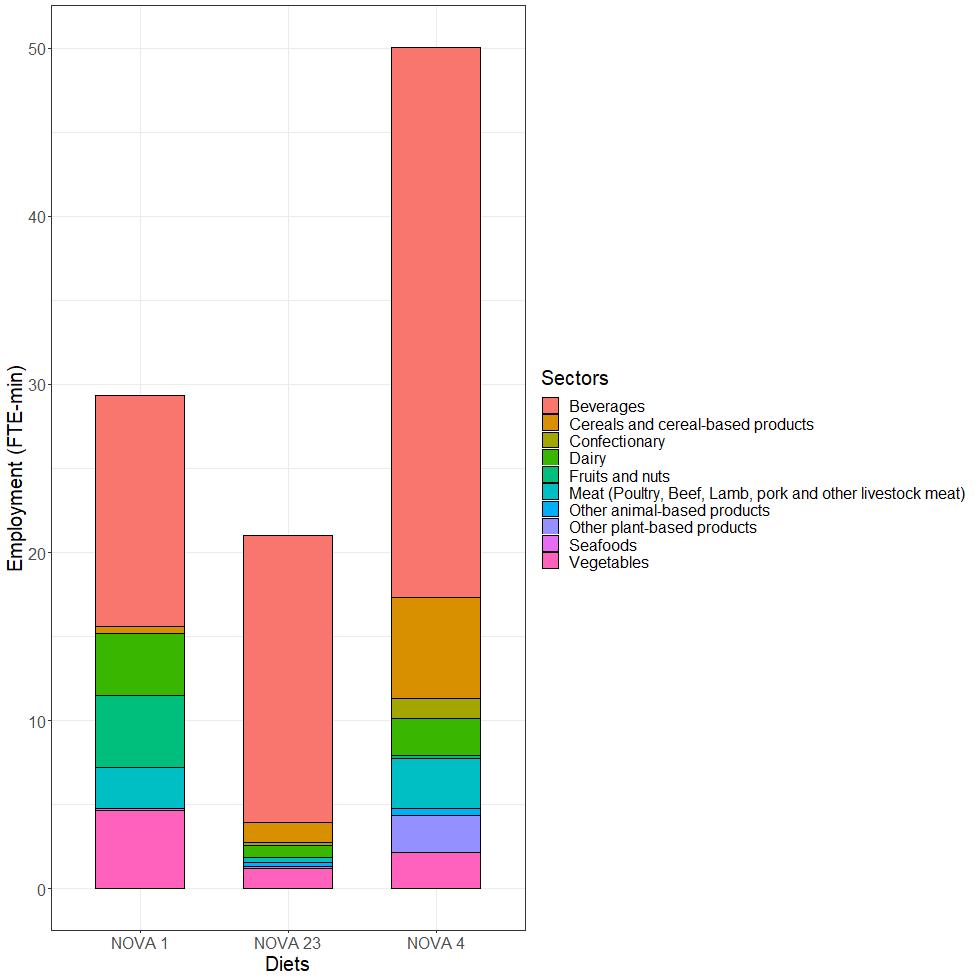


Supplemental Figure 11 - Sector-wise contribution to employment for diets rich in NOVA 1 (>95% energy from NOVA 1), NOVA 23 (>95% energy from NOVA 2 and 3), and NOVA 4 (>95% energy from NOVA 4)
